# Smartphone sensor data estimate alcohol craving in a cohort of patients with alcohol-associated liver disease and alcohol use disorder

**DOI:** 10.1097/HC9.0000000000000329

**Published:** 2023-12-07

**Authors:** Tiffany Wu, Garrick Sherman, Salvatore Giorgi, Priya Thanneeru, Lyle H. Ungar, Patrick S. Kamath, Douglas A. Simonetto, Brenda L. Curtis, Vijay H. Shah

**Affiliations:** 1 Division of Gastroenterology and Hepatology, Mayo Clinic, Rochester, Minnesota, USA; 2 National Institute on Drug Abuse Intramural Research Program, National Institute of Health Baltimore, Maryland, USA; 3 Department of Medicine and Pediatrics, The Brooklyn Hospital Center, Brooklyn, New York, USA; 4 Department of Computer and Information Science, University of Pennsylvania, Philadelphia, Pennsylvania, USA

## Abstract

**Background:**

Sensors within smartphones, such as accelerometer and location, can describe longitudinal markers of behavior as represented through devices in a method called digital phenotyping. This study aimed to assess the feasibility of digital phenotyping for patients with alcohol-associated liver disease and alcohol use disorder, determine correlations between smartphone data and alcohol craving, and establish power assessment for future studies to prognosticate clinical outcomes.

**Methods:**

A total of 24 individuals with alcohol-associated liver disease and alcohol use disorder were instructed to download the AWARE application to collect continuous sensor data and complete daily ecological momentary assessments on alcohol craving and mood for up to 30 days. Data from sensor streams were processed into features like accelerometer magnitude, number of calls, and location entropy, which were used for statistical analysis. We used repeated measures correlation for longitudinal data to evaluate associations between sensors and ecological momentary assessments and standard Pearson correlation to evaluate within-individual relationships between sensors and craving.

**Results:**

Alcohol craving significantly correlated with mood obtained from ecological momentary assessments. Across all sensors, features associated with craving were also significantly correlated with all moods (eg, loneliness and stress) except boredom. Individual-level analysis revealed significant relationships between craving and features of location entropy and average accelerometer magnitude.

**Conclusions:**

Smartphone sensors may serve as markers for alcohol craving and mood in alcohol-associated liver disease and alcohol use disorder. Findings suggest that location-based and accelerometer-based features may be associated with alcohol craving. However, data missingness and low participant retention remain challenges. Future studies are needed for further digital phenotyping of relapse risk and progression of liver disease.

## Graphical abstract

**Figure:**
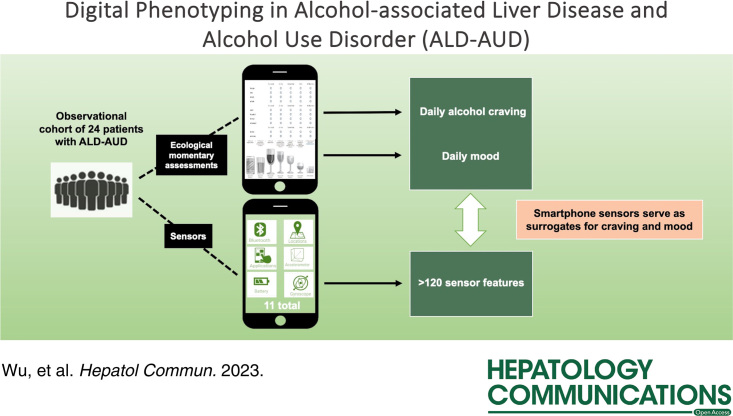

## INTRODUCTION

Sustained abstinence is the most effective strategy to prevent alcohol-associated liver disease (ALD) progression and thereby prolong patient survival.^1,2^ Clinical studies indicate that half of the patients with ALD report alcohol consumption in the past year, and over 30% of individuals hospitalized with ALD report alcohol consumption within 30 days of discharge.^3,4^ A growing body of evidence emphasizes the beneficial impact of treating alcohol use disorder (AUD) in patients with ALD,^5–7^ and treatment of AUD has been recommended as best practice guidelines for ALD care.^8,9^ However, access and utilization of AUD therapy remain limited due to obstacles surrounding perceived stigma, inadequate screening, and a limited supply of addiction resources.^10^

Digital solutions using smartphones offer novel opportunities to overcome many of these barriers and improve access to care. Such solutions provide convenience, data sharing, and delivery of interventions for a wide range of behavioral health conditions, including stigmatized diseases such as mental illness,^11^ smoking,^12^ and alcohol use.^13^ Furthermore, smartphones enable the collection of granular, moment-by-moment measures of behavior with minimal active involvement from users. Information collected from smartphone sensors can identify patterns over prolonged periods as users perform their routine activities. Digital phenotyping refers to the method of quantifying markers of individual behavior as represented through digital technologies.^14^ As a form of personalized medicine, digital phenotyping may improve our understanding of behavioral markers related to disease susceptibility and progression. This knowledge can guide the development of targeted, real-time treatment interventions based on disease phenotype.

Digital phenotyping for patients may provide insight into the relationships between behavior, alcohol relapse, and clinical outcomes of ALD-AUD. As alcohol craving is associated with short-term relapse,^15^ monitoring and management of craving are important in preventing relapse and progression of disease. Therefore, we conducted a pilot study to establish proof-of-concept for digital phenotyping to measure alcohol craving in patients with ALD-AUD. We aimed to assess the feasibility of digital phenotyping in this patient population, describe correlates of craving using data collected from smartphones, and determine power assessment for predicting clinical outcomes in future digital phenotyping studies.

## METHODS

### Patient recruitment and enrollment

This study prospectively recruited a cohort of individuals aged 18 and over with ALD-AUD from the Mayo Clinic in Rochester, Minnesota, between September 2021 and July 2022. Patients were identified and screened for eligibility in the inpatient and outpatient settings using clinical information from the electronic medical record. Patients who responded to recruitment flyers and advertisements posted within the Mayo Clinic campus were also screened for eligibility. Inclusion criteria required a diagnosis of ALD based on the history of regular and excessive alcohol consumption in the absence of other causes of liver disease, with compatible clinical, laboratory, imaging, and histology findings (if a biopsy was performed), as well as a diagnosis of AUD of all severity levels based on history consistent with Diagnostic and Statistical Manual of Mental Disorders-5 diagnostic criteria. Individuals were eligible if they owned a smartphone with cellular data and wireless internet connection and if they were able and willing to provide written informed consent. Exclusion criteria included moderate to severe HE (defined by West Haven score of 3 or higher) and severe psychiatric comorbidity not controlled by pharmacological or behavioral therapy. Power analysis for sample size was not performed in this pilot as this was an objective of the study. All research was conducted in accordance with both the Declarations of Helsinki and Istanbul. All study procedures were approved by the Institutional Review Board at Mayo Clinic, and all participants provided written informed consent.

### Study procedures

In this observational study, participants completed an initial visit where demographic information and medical history related to liver disease and alcohol use were collected by study staff. The participants were then asked to complete a series of assessments for behavioral and psychological characteristics, including depression (Patient Health Questionnaire-9),^16^ anxiety (Generalized Anxiety Disorder),^17^ stress (Perceived Stress Scale),^18^ resilience (Connor–Davidson Resilience Scale-10),^19^ perceived social support,^20^ self-efficacy (General Self-Efficacy Scale),^21^ insight (modified Hanil Alcohol Insight Scale),^22^ readiness to change,^23^ and subjective well-being (Cantril’s Ladder).^24^ Additionally, they completed a baseline Chronic Liver Disease Questionnaire (CLDQ), which systematically provides a measurement of health-related quality of life specific to patients with liver disease and has been used to assess longitudinal change over time.^25^

During the initial visit, the participants downloaded and installed the AWARE application onto their smartphones.^26^ The AWARE application enabled the continuous collection of passive sensor data and delivery of ecological momentary assessments (EMAs) for active data collection on craving, alcohol or substance use, and mood. If hospitalized at the time of study enrollment, EMAs were manually programmed to be delivered after discharge. For 30 days, the participants were instructed to continue their daily activities and routine smartphone usage while keeping AWARE open in the background. After 30 days, they were asked to complete a follow-up visit for the second CLDQ assessment to determine longitudinal change and a timeline follow-back for protocolized assessment of alcohol use during the study period. During the follow-up visit, the participants were instructed to remove the AWARE application from their smartphones, though some chose not to immediately remove it. Initial and follow-up visits were conducted in person or virtually on video. The participants had the ability to call the study staff for assistance if needed. If the participants did not respond to daily EMAs for at least three consecutive days, then the study staff was notified, and the participants were contacted through text or phone reminders. The participants who stopped transmitting data from AWARE or responding to EMAs were deemed to be lost to follow-up. The participants received remuneration based on the time spent in the study, with $30 for completion of each initial and follow-up visit, $1 for each daily EMA, and an additional $10 for 80% EMA completion.

### Data collection and processing

We captured 3 types of data in this study: continuous passive smartphone sensor data, active symptom-based EMAs, and clinical data available through the electronic medical record.

#### Passive data

The AWARE framework is an open-source software package designed for smartphone data collection, consisting of a smartphone application that accesses and transmits sensor data and a server that collects and stores data. For this study, AWARE collected continuous data from eleven sensor streams. Most of these are hardware sensors and components located on most consumer smartphones, such as the accelerometer, global positioning system, or phone screen, as well as software sensors, such as applications, calls, and message logs. For each sensor, AWARE collects raw values sampled with high frequency that amasses millions of data points per participant. No personal identifiers were collected or stored within the AWARE application or server. As data entered a secure server, potentially identifiable keystroke data were automatically deidentified using the state-of-the-art text processing Python package Spacy.^27^

**FIGURE 1:**
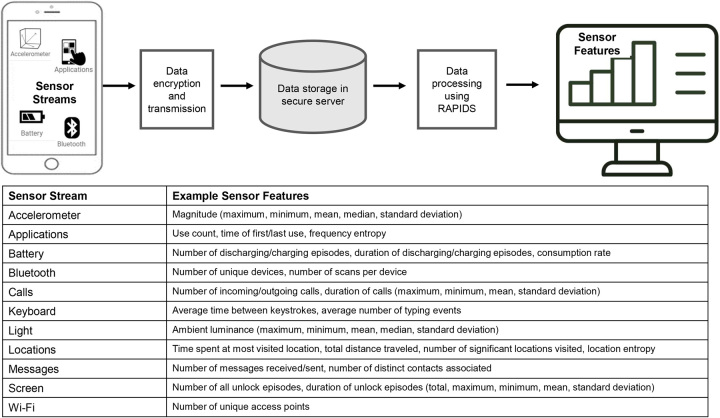
Framework for AWARE data collection and processing.

Raw sensor data were then processed using the Reproducible Analysis Pipeline for Data Streams toolkit into aggregated behavioral sensor features for each sensor stream.^28^ Supplemental A, http://links.lww.com/HC9/A669, lists all processed sensor features included in the analysis. For example, each participant’s location sensor stream is processed into single daily features that provide the number of significant locations visited, time spent, or movement between locations. The sensor stream may also be processed to compute variables such as location entropy, which is based on information theory and quantifies the proportion of time spent at each significant location visited that day.^29^ Figure 1 summarizes the methods of AWARE data collection and processing.

#### Active data

Daily EMA questions surveyed alcohol craving using the 3-item measure of alcohol craving (Brief Alcohol Craving Scale), which has been validated to predict alcohol use the following week (OR = 1.31, 95% CI 1.16–1.47, *p* < 0.001).^15^ The 3 items were summed to create a composite score reflecting total craving intensity (score range 0–12). Participants also responded to daily questions on the number of standard drinks containing alcohol consumed and tobacco or other substances used in the past 24 hours. They were asked to rate various moods (happy, sad, angry, afraid, calm, hopeful, lonely, stressed, bored, anxious, and feeling of social support) on a 5-point Likert scale (Supplemental B, http://links.lww.com/HC9/A669). Daily EMAs were delivered to the participants at 18:00, and they had a 3-hour window to provide a response.

#### Clinical data

We used the electronic medical record to obtain clinical data, including laboratory values (alanine aminotransferase, aspartate aminotransferase, bilirubin, creatinine, sodium, and INR) around the time of the initial and follow-up visits. We also identified clinical events such as death, ED admission or hospitalization, and documentation of alcohol relapse. We examined data up to 90 days after the last participant contact (follow-up visit or earlier if lost to follow-up).

### Statistical analysis

We investigated associations between sensors and EMAs in the cohort. We performed additional subgroup analysis for individuals with a history of alcohol-associated hepatitis (AH) as defined by the criteria from the National Institute on Alcohol Abuse and Alcoholism^30^ and individuals with the status of cirrhosis. As a primary outcome, we examined daily alcohol craving as a continuous variable (score range 0–12). As secondary outcomes, we evaluated changes in mean scores on CLDQ assessment, changes in MELD score, 90-day ED admission or hospitalization, and 90-day alcohol relapse.

We assessed the relationship between alcohol craving and mood using repeated measures correlation through the rmcorr package in R.^31^ The repeated measures correlation uses analysis of covariance to establish the common within-subject association between 2 measures and is therefore well-suited to our longitudinal data. To analyze intraindividual correlations between sensor features and craving, we calculated a standard Pearson correlation coefficient with associated *p*-value using a *t*-test.

All hypothesis tests report statistical significance using an alpha level of 0.05. We use the Benjamini–Hochberg correction for multiple hypothesis testing unless otherwise noted.^32^ This observational cohort study is being reported in line with the Strengthening the Reporting of Observational Studies in Epidemiology guidelines.^33^

## RESULTS

### Baseline characteristics

Twenty-four individuals with ALD-AUD enrolled in the study and completed their initial visits. Table 1 provides the demographic and disease characteristics of the cohort, and Table 2 shows baseline behavioral and psychological traits as characterized by psychometric assessments conducted during the initial visit. Notably, the median age was 49 years (IQR 39.8–57.3), with participants who were predominantly male (70.8%) and White (83.3%). Across ALD stages at the time of enrollment, 6 (25.0%) had steatosis, 2 (8.3%) had advanced fibrosis/compensated cirrhosis, and 15 (62.5%) had decompensated cirrhosis. Thirteen (54.2%) had a history of AH, and the median MELD score across the entire cohort was 10.5 (IQR 8.0–18.0). The median baseline Alcohol Use Disorders Identification Test score was 25, indicating a high likelihood of alcohol dependence (moderate-severe alcohol use disorder), and the mean baseline alcohol craving score was generally reported to be low. Seventeen (70.8%) participants reported a history of receiving psychotherapy-based treatment for AUD, and although this included either residential or outpatient program completion, no participants were actively enrolled in a residential treatment program at the time of study participation.

**TABLE 1:** Baseline demographic and disease-related characteristics for all participants enrolled (n = 24)

Characteristic	Total enrolled (n = 24)
Age in years	49.0 (39.8, 57.3)
Male, n (%)	17 (70.8)
Race, n (%)	
White	20 (83.3)
American Indian	3 (12.5)
Asian	1 (4.2)
Education, n (%)	
High school graduate or GED	6 (25.0)
Some college	6 (25.0)
College graduate	10 (41.7)
Post-college graduate degree	2 (8.3)
Employment, n (%)	
Working for income	13 (54.2)
Retired	3 (12.5)
Unemployed	8 (33.3)
Marital status, n (%)	
Never married	7 (29.2)
Married	10 (41.7)
Separated/divorced	7 (29.2)
Presence of caregivers, n (%)	15 (62.5)
Body mass index	29.9 (26.9, 32.3)
Baseline ALD stage, n (%)	
Steatosis	6 (25.0)
Advanced fibrosis/cirrhosis	2 (8.3)
Decompensated cirrhosis	15 (62.5)
Received transplant	1 (4.2)
History of alcohol-associated hepatitis, n (%)	13 (54.2)
Presence of ascites, n (%)	13 (54.2)
Presence of varices, n (%)	5 (20.8)
Presence of encephalopathy^a^, n (%)	6 (25.0)
Laboratory values	
AST	46.5 (36.8, 75.0)
ALT	30 (26.8, 47.0)
Total bilirubin	1.8 (0.8, 4.0)
Creatinine	0.9 (0.8, 1.0)
INR	1.2 (1.0, 1.4)
Sodium	136.0 (132.8, 139.3)
MELD Score	10.5 (8.0, 18.0)
Baseline AUDIT score^b^	25.0 (11.5, 28.5)
Interval of abstinence before study in days	97.0 (10.8, 178.8)
Family history of AUD, n (%)	19 (79.2)
History of alcohol-related legal issues, n (%)	9 (37.5)
History of psychotherapy-based treatment for AUD^c^, n (%)	17 (70.8)
Active pharmacotherapy for AUD^d^, n (%)	6 (25.0)
History of comorbid psychiatric disorders, n (%)	
Depression	11 (45.8)
Bipolar disorder	2 (8.3)
Anxiety	11 (45.8)
Post-traumatic stress disorder	2 (8.3)
Active pharmacotherapy for comorbid psychiatric disorders, n (%)	12 (50.0)
Nicotine use disorder, n (%)	15 (62.5)
Cannabis use disorder, n (%)	7 (29.2)
Opioid or other substance use disorder, n (%)	2 (8.3)

All continuous values are provided with median (IQR).

a Defined as West Haven Grade less than 3.

b One observation is missing and not included in the analysis.

c Defined as completion of either residential or outpatient treatment program.

d Defined as use of FDA-approved medications for AUD, including acamprosate, naltrexone, and disulfiram.

Abbreviations: ALD, alcohol-associated liver disease; AUD, alcohol use disorder; AUDIT, Alcohol Use Disorders Identification Test.

**TABLE 2:** Baseline behavioral and psychological characteristics for all participants enrolled (n = 24)

Questionnaire	Total enrolled (n = 24)
Patient Health Questionnaire (PHQ-9) Total Score, mean (SD) Score range 0–27	6.7 (5.1)
Generalized Anxiety Disorder (GAD-7) Total Score, mean (SD) Score range 0–21	6.0 (5.2)
Perceived Stress Scale Total Score, mean (SD) Score range 0–40	15.4 (6.0)
Connor–Davidson Resilience Scale (CD-RISC)-10 Total Score, mean (SD) Score range 0–40	28.4 (5.1)
Perceived Social Support (F-SozU K-6) Total Score, mean (SD) Score range 6–30	23.0 (4.8)
General Self-Efficacy Scale Total Score, mean (SD) Score range 8–40	29.5 (4.8)
Insight Scale: Agree response, n (%)	
I find many problems with my drinking.	19 (79.2)
I can control drinking any time if I want to.	9 (37.5)
All my problems can be solved only when I quit drinking.	10 (41.7)
My drinking did no harm to any member of the family.	4 (16.7)
Readiness to change Item Score, mean (SD) Score range 0–10	
Importance	9.1 (2.3)
Confidence	8.5 (1.7)
Readiness to change	9.4 (1.7)
Subjective Well-being Item Score, mean (SD) Score range 0–10	7.0 (1.8)
Chronic Liver Disease Questionnaire Item Score, mean (SD) Score range 1–7	
Abdominal symptoms domain (1, 5, 17)	4.9 (1.6)
Fatigue domain (2, 4, 8, 11, 13)	3.7 (1.3)
Systemic domain (3, 6, 21, 23, 27)	4.7 (1.2)
Activity domain (7, 9, 14)	4.3 (1.4)
Emotional function domain (10, 12, 15, 16, 19, 20, 24, 26)	4.6 (1.0)
Worry domain (18, 22, 25, 28, 29)	4.1 (1.5)
Overall score	4.4 (1.0)
Brief Alcohol Craving Scale Total Score, mean (SD) Score range 0–12	2.6 (2.6)

Abbreviations: CD-RISC, Connor–Davidson Resilience Scale; GAD-7, Generalized Anxiety Disorder-7; PHQ-9, Patient Health Questionnaire-9

### Study feasibility and retention

During the study period, 163 individuals were approached for screening. Of the 163 total screened, 24 (14.7%) enrolled in the study and 139 (85.3%) did not. Of the individuals who did not enroll, 98 (70.5%) were not interested, 22 (15.8%) reported having incompatible technology, and 19 (13.7%) stopped responding prior to enrollment. Among the 24 participants who enrolled in the study, 12 had adequate AWARE and EMA data and were deemed to have completed all study components, thereby representing a 50% retention rate in this study. A flow diagram of recruitment and retention is included in Supplemental C, http://links.lww.com/HC9/A669.

Participants were withdrawn early for multiple reasons. Five reported technical issues with the AWARE application, including 3 people who stated that it caused problems with other smartphone functions and found the application to be burdensome, one who lost cellular service after enrollment, and one who had difficulty with application installation despite multiple attempts to download. Two participants lost interest in the study and chose to withdraw. An additional 4 individuals lost contact despite attempts to follow up from study staff, and one individual died from complications due to decompensated cirrhosis before completing any EMAs.

To understand the practical challenges in study recruitment and retention among patients with ALD-AUD, we compared the baseline characteristics of the individuals who completed the study and those who did not. As described, participants were withdrawn for various reasons, including both technological issues and loss of interest. Supplemental D, http://links.lww.com/HC9/A669, provides comparative differences between the demographic and disease characteristics, along with the behavioral and psychological traits at baseline. Importantly, there were no significant differences between those who completed the study and those who did not. This suggests that the findings may be generalizable, and the risk of dropping out of the study may not be attributed to differences in factors such as baseline disease severity, insight, or readiness to change.

### Data availability

Among the entire cohort, 12 (50.0%) participants had AWARE sensor data available for at least 30 days, and 3 (12.5%) did not share any AWARE data after enrollment. The median number of days that AWARE data were transmitted was 34.5 (IQR 28.8) per participant. More sensor types were available on average from smartphones with Android compared to Apple iPhone Operating System (8.4 vs. 4.7 sensor types). Distribution of sensor data availability by the participant is provided in Supplemental E, http://links.lww.com/HC9/A669, which illustrates that the three most common sensors available were accelerometer, screen, and Wi-Fi network.

The number of EMAs completed corresponds to the number of per-participant observations for daily craving score, standard alcohol-containing drinks consumed, tobacco or other substance use, and mood. Twelve participants completed at least 20 days of EMAs along with the follow-up visit, and 3 completed < 20 days of EMAs but were withdrawn from the study before completing the follow-up visit. A total of 7 did not complete any EMAs before study withdrawal. All participants supplied AWARE data every day that they responded to EMAs. Some participants chose to supply additional days of AWARE data after EMAs ceased (mean 14.6 d), even after being reminded to remove the AWARE application from their phones.

Clinical events, including death, ED admission or hospitalization, and documented alcohol relapse, were followed for up to 90 days after the last participant contact. Overall, 1 participant died from disease complications unrelated to the study, 9 participants had presented to the ED or were hospitalized, and 7 participants had documented alcohol relapse within 90 days. Table 3 summarizes clinical events as grouped by the total days of EMA completion (20 days, <20 d, or none).

**TABLE 3:** Clinical outcomes at 90 days for participants, grouped by degree of study completion

Outcome	Completed 20 days of EMAs (n = 12)	Completed < 20 days of EMAs (n = 5)	Completed 0 days of EMAs (n = 7)	Total (n = 24)
90-day death, n (%)				
Yes	0	0	1 (14.3)	1 (4.2)
No	9 (75.0)	4 (80.0)	6 (85.7)	19 (79.2)
Unknown	3 (25.0)	1 (20.0)	0	4 (16.7)
90-day ED admission or hospitalization, n (%)				
Yes	4 (33.3)	1 (20.0)	4 (57.1)	9 (37.5)
No	5 (41.7)	3 (60.0)	3 (42.9)	11 (45.8)
Unknown	3 (25.0)	1 (20.0)	0	4 (16.7)
90-day relapse, n (%)				
Yes	2 (16.7)	1 (20.0)	4 (57.1)	7 (29.2)
No	7 (58.3)	3 (60.0)	3 (42.9)	13 (54.2)
Unknown	3 (25.0)	1 (20.0)	0	4 (16.7)

Abbreviations: EMA, ecological momentary assessment.

### Correlates of craving and mood in ALD-AUD

Among the participants with 20 days of observations for daily craving score, 8 had at least 2 days of change in craving score. Since the change in craving score was our primary end point, we examined relationships between passive and active data with craving variation (Supplemental F, http://links.lww.com/HC9/A669).

**FIGURE 2:**
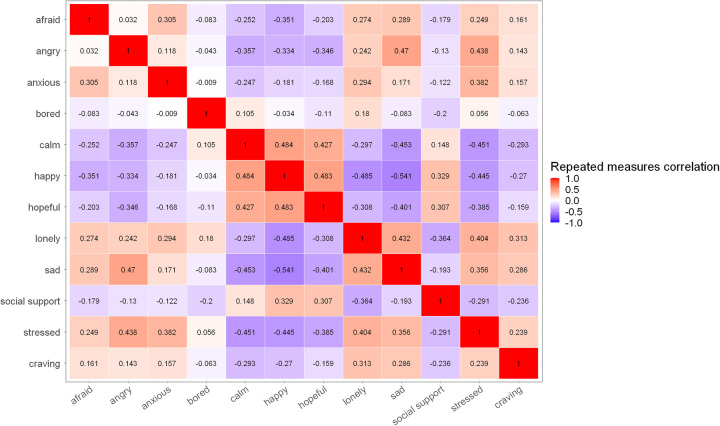
Matrix of repeated measures correlations among craving and moods, as collected through EMA responses. Abbreviation: EMA, ecological momentary assessments.

When assessing the correlation between craving and moods from actively collected EMAs (Table 4), craving was positively correlated with negative moods, including loneliness (*r* = 0.313, *p* < 0.001), sadness (*r* = 0.286, *p* <0.001), stress (*r* = 0.239, *p* = 0.002), fear (*r* = 0.161, *p* = 0.038), and anxiety (*r* = 0.157, *p* = 0.038). Thus, a more negative mood was associated with more craving intensity. Interestingly, anger (*r* = 0.143, *p* = 0.054) and boredom (*r* = −0.063, *p* = 0.390) were not significantly correlated with craving. Conversely, craving was negatively correlated with positive moods, including calmness (*r* = −0.293, *p* < 0.001), happiness (*r* = −0.270, *p* < 0.001), social support (*r* = −0.236, *p* = 0.002), and hope (*r* = −0.159, *p* = 0.038). Figure 2 illustrates the correlation matrix across craving and all moods.

**TABLE 4:** CORRELATES of craving and mood, by ecological momentary assessment responses

Mood	Correlation coefficient (r)	*p*	95% CI	Adjusted *p*
Lonely	0.313	<0.001	(0.178, 0.437)	<0.001
Sad	0.286	<0.001	(0.149, 0.412)	<0.001
Stressed	0.239	0.001	(0.100, 0.370)	0.002
Afraid	0.161	0.026	(0.019, 0.298)	0.038
Anxious	0.157	0.031	(0.014, 0.293)	0.038
Angry	0.143	0.049	(0.000, 0.280	0.054
Bored	−0.063	0.390	(−0.204, 0.081)	0.390
Hopeful	−0.159	0.028	(−0.295, −0.016)	0.038
Social support	−0.236	0.001	(−0.367, −0.096)	0.002
Happy	−0.270	<0.001	(−0.398, −0.132)	<0.001
Calm	−0.293	<0.001	(−0.418, −0.156)	<0.001

**FIGURE 3:**
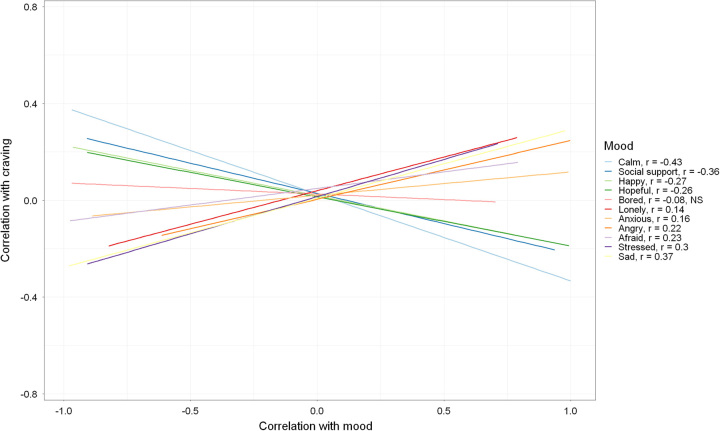
Sensors associated with craving versus sensors associated with individual moods. Each mood is depicted with a line of best fit, showing the trend between sensor correlations with mood and sensor correlations with craving. Negative moods show a positive slope, indicating that sensors that correlate more highly with negative moods tend to also correlate more highly with craving. Positive moods show a negative slope, indicating that sensors that correlate more highly with positive moods tend to correlate more negatively with craving. The only mood that does not exhibit a significant line of best fit is boredom. The figure shows that, in aggregate, sensors act as a proxy for mood in relation to craving.

A similar directionality of correlations between craving and mood was found using passive sensor data alone. When establishing a trend between sensor correlations with mood and sensor correlations with craving, the sensors that correlated more highly with negative mood tended to correlate more negatively with craving, while the sensors that correlated more highly with positive moods tended to correlate more negatively with craving. For instance, sensors associated with craving also tended to associate with sadness (*r* = 0.374, *p* < 0.001), while the same sensors showed the inverse tendency with calmness (*r* = −0.432, *p* < 0.001). Figure 3 illustrates the plot of sensor correlations between craving and moods. Sensor relationships were significantly associated with all moods except for boredom. This shows in aggregate that sensors act as a surrogate for mood in relationship to craving.

### Sensors as digital phenotypes of ALD-AUD

**FIGURE 4:**
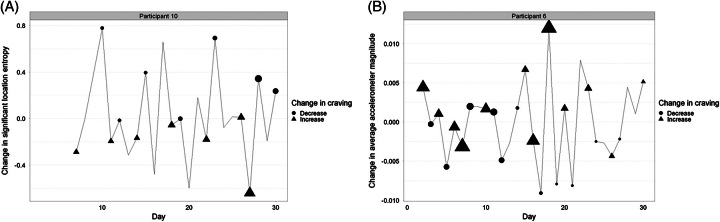
A shows a within-person analysis of significant location entropy vs. daily craving for one participant (*r* = −0.583, *p* = 0.004). Each point reflects the change in significant location entropy on a given day, relative to the previous day. The shape of each point corresponds to the direction of change in alcohol craving (triangle = increase, circle = decrease), and the size of each point corresponds to the degree of that change (larger size indicates greater change in craving). B shows a within-person analysis of change in average accelerometer magnitude versus daily craving for 1 participant (*r* = 0.629, *p* < 0.001). Each point reflects the change in average accelerometer magnitude on a given day, relative to the previous day. The shape of each point corresponds to the direction of change in alcohol craving (triangle = increase, circle = decrease), and the size of each point corresponds to the degree of that change (larger size indicates greater change in craving).

To investigate the relationship between craving and sensor data, we examined sensor activity to understand personalized digital phenotypes. We used individual-level data to analyze the change in sensor features and the change in daily craving. In this analysis, we identified specific phenotypes related to sensor features, which measure participant mobility. One individual phenotype showed a strong relationship between craving and location entropy (*r* = −0.583, *p* = 0.004) (Figure 4A). Over 30 days, most days with positive change in location entropy (positive value on the y-axis) were associated with a decrease in craving score compared to the previous day. Days with a negative change in location entropy (negative value on the y-axis) were associated with an increase in craving score compared to the previous day. Therefore, this participant’s behavioral pattern revealed that they experienced higher cravings on the days when their time was primarily concentrated in a smaller number of locations.

Another individual phenotype showed a relationship between craving and average accelerometer magnitude (*r* = 0.629, *p* < 0.001; Figure 4B). Over 30 days, days with an increase in the average accelerometer magnitude (ie, detected movement) were associated with an increase in craving score compared to the previous day. Similarly, days with a decrease in accelerometer magnitude were associated with a decrease in craving score compared to the previous day. While the accelerometer sensor specifically measures smartphone acceleration, including the force of gravity, data are transformed into information that is interpreted as relative movement over time.

### Sensors by subgroups of alcohol-associated hepatitis and cirrhosis

Individuals were classified into subgroups of known clinical phenotypes of disease to assess for differences in sensor activity over time. Specifically, the history of AH and the presence of cirrhosis were evaluated. Means for each sensor feature were calculated, estimates were compared using a standard two-sided Student *t*-test for significance, and *p* values were corrected using the Benjamini–Hochberg procedure. For AH, 13 (54.2%) participants had a history or current AH at the time of enrollment and 11 (45.8%) did not. There were no significant differences in sensor activity between groups.

We investigated whether the associations between sensor features and craving score differed depending on the clinical disease phenotype. We calculated the repeated measures correlation between sensor values and craving, producing for each sensor one correlation coefficient for those with ALD phenotype presence and one for those without, and took the difference. Finally, we performed a permutation test to calculate the statistical significance of the observed difference in correlations. Due to the small sample size and uneven sampling of sensor types across participants, we had insufficient data to calculate meaningful correlation coefficients. However, relatively low *p* values were calculated for certain accelerometer features when comparing AH groups (Supplemental G, http://links.lww.com/HC9/A669). Given the suggested phenotype linking accelerometer data and craving, future work should pay particular attention to ensuring an adequate sample of accelerometer data for further exploration.

For cirrhosis status, 18 (75.0%) participants had been diagnosed with cirrhosis before enrollment. This included both compensated and decompensated cirrhosis (including 1 participant who received liver transplant for decompensated cirrhosis < 4 mo before enrollment). When comparing subgroups of cirrhosis status, there were also no significant differences in sensor activity between groups. Similar repeated measures correlation calculations were also limited by small sample size and uneven sampling.

### Predictors of 30-day and 90-day outcomes

Across the participants who completed the follow-up visit, we evaluated the relationship between the change in CLDQ score against EMA and average sensor readings. We found that the mean change in CLDQ score from baseline to follow-up improved overall and within each individual domain (Supplemental H, http://links.lww.com/HC9/A669). Overall, the score improved from a mean of 4.1 (SD 0.8) at baseline to a mean of 4.5 (SD 0.8) at follow-up, where a higher numerical score (range 0–7) indicates a lower symptom burden.

There were no statistically significant correlations between the change in CLDQ score and mood measures after 30 days, although there was a moderately strong relationship between CLDQ change and hopefulness (Supplemental I, http://links.lww.com/HC9/A669). When we explored the correlations between the change in CLDQ and sensors, there were only 2 sensors with significant correlations on unadjusted significance testing (keyboard change in text length, keyboard session count), and these were based on small sample sizes of three individuals who had all keyboard data (Supplemental J, http://links.lww.com/HC9/A669).

No significant correlation was identified between moods or average sensor readings and change in MELD score from baseline to follow-up. This was expected given the short duration of follow-up after 30 days. However, when we examined alcohol craving as a predictor, we found that craving had a strong but nonsignificant correlation with the number of drinks consumed (*r* = 0.635, *p* = 0.091) and nicotine use (*r* = −0.580, *p* = 0.131) during monitoring.

We also explored whether relapse or hospital readmission at 90 days was associated with sensor features. In each case, 17 participants had available sensor data with associated relapse or readmission data. We computed the mean value of each sensor feature across the study period. We then calculated unpaired *t*-tests for each sensor to evaluate sensor differences between those who were and were not readmitted and, analogously, those who did and did not relapse. On average, we found that accelerometer magnitude was higher among those who relapsed, and the time of the first daily text message was earlier among individuals readmitted to the hospital. Although these features were not statistically significant after applying the Benjamini–Hochberg correction, they provide a useful focal point for future studies.

## DISCUSSION

Our early experience using the AWARE application to understand the digital phenotypes of patients with ALD-AUD advances personalized care in digital health care delivery. This study demonstrates that digital phenotyping is a feasible method for disease monitoring and prognostication in this population. We showed that smartphone sensors may serve as surrogates for alcohol craving and mood and could potentially predict clinical outcomes such as relapse or readmission. Our findings complement published work using similar sensor data to detect active drinking,^34^ along with future cravings for tobacco^35^ and other substances.^36^ Digital phenotyping has also been investigated among patients with schizophrenia^37–39^ and bipolar disorder^40^ to assess symptoms or transitions between disease states and to predict stress or lifestyle behaviors among healthy populations.^41–43^ Here, the collection and analysis of longitudinal markers of behavior through smartphone data establish the viability of digital phenotyping in ALD-AUD as a promising application for personalized care in liver disease.

Disease heterogeneity in ALD-AUD makes a uniform approach to treatment impracticable because patients’ needs are unique. Digital phenotyping addresses this disease heterogeneity by capturing specific attributes of the individual. In this study, we found signals between sensor features, such as location entropy and accelerometer magnitude, and alcohol craving on individual-level analysis. By identifying significant patterns in individual behavior, this data-driven approach demonstrates that digital phenotyping can address the heterogeneity of ALD-AUD expression. As a comparator to these novel behavioral phenotypes, we explored sensor activity in subgroups using known clinical phenotypes of liver disease such as AH and cirrhosis. Although there were no significant differences in sensor features between these groups, the study was not powered to detect these differences. Nevertheless, new digital phenotypes could measure disease characteristics not previously known or accounted for, transforming our understanding of pathogenesis in ALD-AUD. This can impact every stage in patient care, from identifying the risk of developing liver disease among heavy drinkers to predicting disease progression among those already diagnosed with ALD.

A major advantage of digital phenotyping is scalability and longitudinal follow-up using ubiquitous technologies like smartphones. The AWARE application allows data collection from individual smartphones without active user engagement (passive data) and data from prompted surveys regarding in-the-moment measures (active data), such as alcohol craving. When assessing active data from EMAs alone, we found that daily mood was significantly associated with alcohol cravings. Specifically, craving was positively correlated with negative moods like loneliness, sadness, stress, fear, and anxiety, while craving was negatively correlated with positive moods like calmness, happiness, supportiveness, and hope. Many of these correlations are congruent with existing knowledge on the impact of mood-related influences on alcohol craving.^44,45^ After incorporating passive sensor data, we found that across all sensors, sensor association with craving was significantly correlated with sensor association with moods. This relationship suggests that sensors may be used as digital markers to monitor the risk of progression in ALD-AUD. This can be done at scale, remotely, and with minimal patient involvement. Digital phenotyping thus offers a novel model of care delivery, such as personalized interventions through smartphones based on real-time data from individual phenotypes.^11,13^

Finally, this study has informed the design of future studies in multiple ways. First, our practical experience with enrollment and participant follow-up has provided valuable insight into the feasibility of digital phenotyping studies in ALD-AUD. During enrollment, a lack of interest in participation contributed to a low enrollment rate. Another reason was related to smartphone incompatibility with the study application; however, this may be less of a barrier as improvements are made to increase application functionality across different smartphone models. During the study period, the retention rate for completing the study was 50%. There were no significant differences in demographic, disease, behavioral, and psychological traits between the 12 participants who completed the study and the 12 who withdrew early. Therefore, despite a low retention rate, the risk for early withdrawal may not be attributed to such factors. Second, this study has contributed useful information to power future studies using sensors to prognosticate clinical outcomes. Here, we tested the use of EMAs and sensors to predict liver-related quality of life as measured by change in CLDQ. While the correlation between mood reported by EMAs and sensors was not significant after adjusting for multiple sensor features, the results provide a reasonable estimate of target effect size for future power analyses. Third, we gathered insights on the viability of AWARE application use and data collection on individual smartphones. Despite following standardized procedures for application installation, we still noted inconsistent data transmittal across individuals. We identified differences between Android and Apple iPhone operating systems, yet there was likely additional variance based on smartphone models, which could not be captured. Finally, we gained technical experience in application configuration and learned that data sparsity must be accounted for in both study recruitment and data analysis.

As such, there are limitations to this study. In this cohort, the median duration of abstinence before study enrollment was around 3 months. The journey from early to sustained abstinence may vary for each patient, and accordingly, results may not be generalizable to all durations of abstinence. Similarly, baseline evaluation indicated low alcohol craving and high scores of individual readiness to change, insight, and self-efficacy. Increased insight may reflect self-selection bias for study participation, which cannot be generalizable to the broader population with AUD. While these characteristics may limit the interpretation of study findings, this underscores disease heterogeneity in AUD, which may be further heightened by individual phenotypes of behavior as well as individual readiness for change. As a result, this highlights the opportunity for digital phenotyping to inform personalized therapy using factors related to addiction. The small sample size was likely underpowered to detect significant predictors of clinical outcomes, including CLDQ, MELD score, ED or hospital admission, or relapse at 90 days. Since alcohol craving is associated with short-term relapse, we anticipate that larger studies may show significant associations between digital phenotypes and readmission or relapse, liver decompensation, or mortality among patients with ALD-AUD. Data missingness due to temporary interruptions (eg, if the phone is turned off or the operating system halts data collection) also limited the available data for analysis. The combination of missing data with limited study retention further highlights the challenges—and critical need—to develop new approaches to improve patient engagement in ALD-AUD.

Digital phenotyping must consider ethical concerns for privacy and data protection. The novel types of data and analytics in digital phenotyping may produce sensitive health information that is not adequately addressed under current ethical and regulatory frameworks. It is critical that personal and biometric data, along with the potential consequences of health-related inferences drawn from this data, are safeguarded so that their use will not create disparities or harm marginalized individuals from different racial, socioeconomic, or stigmatized populations. Furthermore, appropriate transparency and consent procedures will need to inform how data are collected and used. A major challenge may include explaining real-time behavior versus prediction of future risk. Interpreting predictions from digital phenotyping will need to be performed cautiously without negatively impacting care delivery. Further engagement with key stakeholders and regulatory agencies to address these issues will be essential as research continues in this field.

In conclusion, our study suggests that smartphone sensors may serve as surrogates for behavioral markers and classify new disease-related phenotypes in ALD-AUD. Larger studies are needed to understand the relationships between sensor features and outcomes such as relapse, decompensation, or death. Data missingness and participant retention must be addressed. However, these findings serve as a foundation for future studies that are needed to validate the utility of digital phenotypes across varied geographic, socioeconomic, and cultural backgrounds.

## Supplementary Material
