# Supplementary material for: Smartphone sensor data estimate alcohol craving in a cohort of patients with alcohol-associated liver disease and alcohol use disorder
Source: Hepatol Commun. 2023 Dec 7;7(12):e0329. doi: 10.1097/HC9.0000000000000329 (PMC10984664; doi:10.1097/HC9.0000000000000329)
Supplement: Supplementary file 1 [file hc9-7-e0329-s001.docx]

**Supplemental Digital Content**

A: List of all AWARE sensors collected and associated RAPIDS features computed. Feature descriptions are taken largely verbatim from the RAPIDS documentation (https://www.rapids.science/1.9/).

| **Sensor** | **Feature Description** |
| --- | --- |
| Accelerometer | The maximum magnitude of acceleration |
| Accelerometer | The minimum magnitude of acceleration. |
| Accelerometer | The average magnitude of acceleration. |
| Accelerometer | The median magnitude of acceleration. |
| Accelerometer | The standard deviation of acceleration. |
| Applications | Number of times an app in any category was used. |
| Applications | Number of times an app in the email category was used. |
| Applications | Number of times an app in the social media category was used. |
| Applications | Number of times an app in the dating category was used. |
| Applications | Number of times an app in the social category was used. |
| Applications | Number of times an app in the entertainment category was used. |
| Applications | Number of times Twitter was used. |
| Applications | Number of times Facebook was used. |
| Applications | Number of times the user’s most used app was used. |
| Applications | Time of first use of any app. |
| Applications | Time of first use of an email app. |
| Applications | Time of first use of a social media app. |
| Applications | Time of first use of a dating app. |
| Applications | Time of first use of a social app. |
| Applications | Time of first use of an entertainment app. |
| Applications | Time of first use of Twitter. |
| Applications | Time of first use of Facebook. |
| Applications | Time of first use of the user’s most used app. |
| Applications | Time of last use of any app. |
| Applications | Time of last use of an email app. |
| Applications | Time of last use of a social media app. |
| Applications | Time of last use of a dating app. |
| Applications | Time of last use of a social app. |
| Applications | Time of last use of an entertainment app. |
| Applications | Time of last use of Twitter. |
| Applications | Time of last use of Facebook. |
| Applications | Time of last use of the user’s most used app. |
| Applications | The entropy of the apps used (the more apps were used, the higher the entropy). |
| Applications | The entropy of the email apps used (the more apps were used, the higher the entropy). |
| Applications | The entropy of the dating apps used (the more apps were used, the higher the entropy). |
| Applications | The entropy of the social apps used (the more apps were used, the higher the entropy). |
| Battery | The total duration of all discharging episodes. |
| Battery | The total duration of all charging episodes. |
| Battery | Number of battery charging episodes. |
| Battery | Number of discharging episodes. |
| Battery | The average of all episodes’ consumption rates. |
| Battery | The highest of all episodes’ consumption rates. |
| Bluetooth | Number of scans from the devices sensed during a time segment instance. The more scans a Bluetooth device has the longer it remained within range of the participant’s phone |
| Bluetooth | Number of unique Bluetooth devices sensed as identified by their hardware addresses. |
| Bluetooth | Number of scans of the most scanned Bluetooth device. |
| Calls | Number of missed calls that occurred. |
| Calls | Number of distinct contacts that are associated with missed calls. |
| Calls | The time in hours from midnight that the first call was missed. |
| Calls | The time in hours from midnight that the last call was missed. |
| Calls | The number of missed calls of the most frequent contact throughout the monitored period. |
| Calls | Number of incoming calls. |
| Calls | Number of distinct contacts that are associated with incoming calls. |
| Calls | The mean duration of all incoming calls. |
| Calls | The sum of the duration of the incoming calls. |
| Calls | The duration of the shortest incoming call. |
| Calls | The duration of the longest incoming call. |
| Calls | The standard deviation of the duration of the incoming calls. |
| Calls | The mode of the duration of all incoming calls. |
| Calls | The estimate of the Shannon entropy for the duration of the incoming calls. |
| Calls | The time in minutes between midnight and the first incoming call. |
| Calls | The time in minutes between midnight and the last incoming call. |
| Calls | The number of incoming calls of the most frequent contact throughout the monitored period. |
| Calls | Number of outgoing calls. |
| Calls | Number of distinct contacts that are associated with outgoing calls. |
| Calls | The mean duration of all outgoing calls. |
| Calls | The sum of the duration of all outgoing calls. |
| Calls | The duration of the shortest outgoing call. |
| Calls | The duration of the longest outgoing call. |
| Calls | The standard deviation of the duration of the outgoing calls. |
| Calls | The mode of the duration of the outgoing calls. |
| Calls | The estimate of the Shannon entropy for the duration of the outgoing calls. |
| Calls | The time in minutes between midnight and the first outgoing call. |
| Calls | The time in minutes between midnight and the last outgoing call. |
| Calls | The number of outgoing calls of the most frequent contact throughout the monitored period. |
| Keyboard | The average time between keystrokes measured in milliseconds. |
| Keyboard | Length of the last text in characters of the sentence(s) contained in the typing text box of any app. |
| Keyboard | Number of times a keyboard typing or swiping event changed the length of the current text in exactly one more character. |
| Keyboard | Average length of all sessions. |
| Keyboard | Number of times a keyboard typing or swiping event changed the length of the current text to more than one character. |
| Keyboard | Average number of typing events across all sessions. |
| Keyboard | Number of times a keyboard typing or swiping event changed the length of the current text in exactly one fewer character. |
| Keyboard | Number of typing sessions in a time segment. |
| Keyboard | Length in characters of the longest sentence(s) contained in the typing text box of any app. |
| Keyboard | Number of times a keyboard typing or swiping event changed the length of the current text to less than one fewer character. |
| Light | Number light sensor rows recorded. |
| Light | The maximum ambient luminance. |
| Light | The minimum ambient luminance. |
| Light | The average ambient luminance. |
| Light | The median ambient luminance. |
| Light | The standard deviation of ambient luminance. |
| Locations | Time spent at home in minutes. Home is the most visited significant location between 8 pm and 8 am, including any pauses within a 200-meter radius. |
| Locations | Total distance traveled over a day (flights). |
| Locations | The Radius of Gyration (rog) is a measure in meters of the area covered by a person over a day. A centroid is calculated for all the places (pauses) visited during a day, and a weighted distance between all the places and that centroid is computed. The weights are proportional to the time spent in each place. |
| Locations | The maximum diameter is the largest distance between any two pauses. |
| Locations | The maximum distance from home in meters. |
| Locations | The number of significant locations visited during the day. Significant locations are computed using k-means clustering over pauses found in the whole monitoring period. The number of clusters is found iterating k from 1 to 200 stopping until the centroids of two significant locations are within 400 meters of one another. |
| Locations | Mean length of all flights. |
| Locations | Standard deviation of the length of all flights. |
| Locations | Mean duration of all flights. |
| Locations | The standard deviation of the duration of all flights. |
| Locations | The fraction of a day spent in a pause (as opposed to a flight) |
| Locations | Shannon’s entropy measurement is based on the proportion of time spent at each significant location visited during a day. |
| Locations | A continuous metric quantifying a person’s circadian routine that can take any value between 0 and 1, where 0 represents a daily routine completely different from any other sensed days and 1 a routine the same as every other sensed day. |
| Locations | Same as above but computed separately for weekends and weekdays. |
| Messages | Number of messages from the contact with the most received messages during a day throughout the whole study period for each participant. |
| Messages | The number of received messages. |
| Messages | The number of distinct contacts that are associated with received messages. |
| Messages | Number of minutes between midnight and the first received message. |
| Messages | Number of minutes between midnight and the last received message. |
| Messages | The number of messages from the contact with the most sent messages during a day throughout the whole study period for each participant. |
| Messages | The number of sent messages. |
| Messages | The number of distinct contacts that are associated with sent messages. |
| Messages | Number of minutes between midnight and the first sent message. |
| Messages | Number of minutes between midnight and the last sent message. |
| Screen | Number of all unlock episodes |
| Screen | Total duration of all unlock episodes. |
| Screen | Longest duration of any unlock episode. |
| Screen | Shortest duration of any unlock episode. |
| Screen | Average duration of all unlock episodes. |
| Screen | Standard deviation duration of all unlock episodes. |
| Screen | Minutes until the first unlock episode. |
| Wifi | Number of scanned WiFi access points connected. An access point can be detected multiple times over time and these appearances are counted separately |
| Wifi | Number of unique access points connected, as identified by their hardware address |
| Wifi | Number of scans of the most connected access point across the whole monitoring period. |
| Wifi | Number of scanned WiFi access points visible. An access point can be detected multiple times over time and these appearances are counted separately. |
| Wifi | Number of unique access points visible, as identified by their hardware address. |
| Wifi | Number of scans of the most scanned access point across the whole monitoring period |

B: Daily EMA questions.

1.
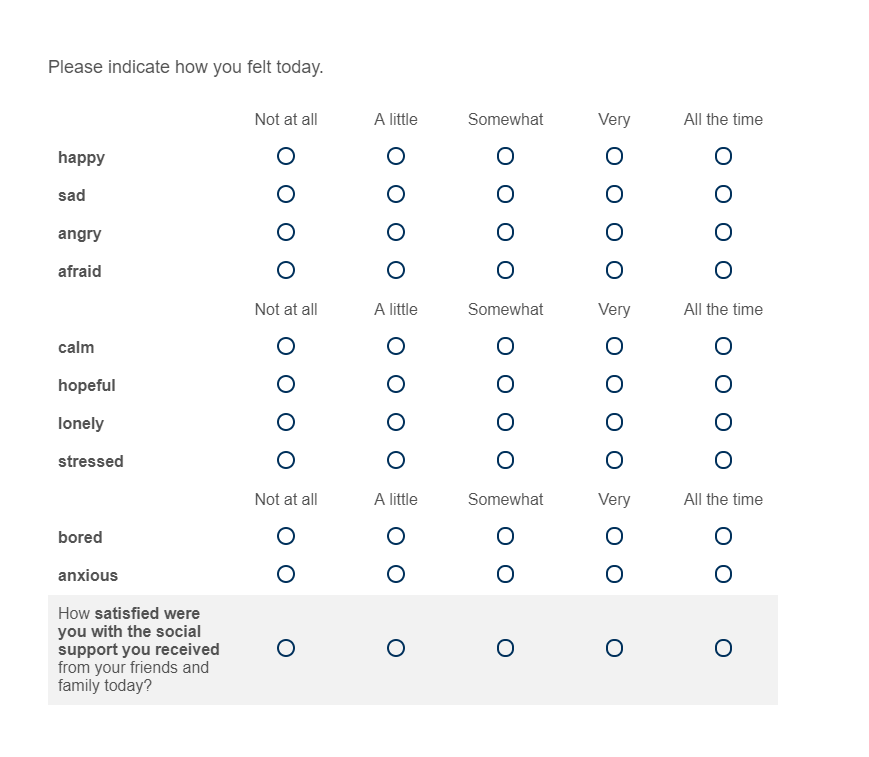

2. How many standard alcoholic drinks did you consume in the past 24 hours? Please note a standard drink is any drink containing 0.6 fluid ounces or 14 grams of pure alcohol:

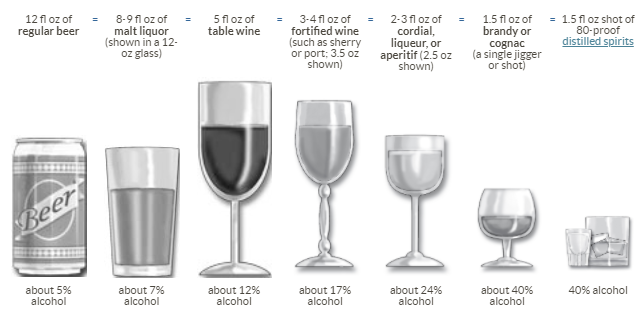

3. How strong is the desire to consume alcoholic beverages?
   1. 0 = Not at all
   2. 1 = A little
   3. 2 = Somewhat
   4. 3 = Very
   5. 4 = All the time
4. Please rate how strong your desire to drink has been when something in the environment has reminded you of drinking (examples: seeing a beer ad, walking past the liquor section in the grocery store).
   1. 0 = Not at all
   2. 1 = A little
   3. 2 = Somewhat
   4. 3 = Very
   5. 4 = All the time
5. Please imagine yourself in the environment in which you previously drank alcohol (for example, a bar, a restaurant, or a particular room where you live). If you were in this environment today and if it were the time of day that you typically drank, what is the likelihood that you would drink today?
   1. 0 = Not at all
   2. 1 = A little
   3. 2 = Somewhat
   4. 3 = Very
   5. 4 = All the time
6. Did you use cigarettes or any drugs in the past 24 hours? If yes, check all that apply:
   1. Cigarettes/nicotine/tobacco/vape juice
   2. Cannabis
   3. Cocaine
   4. Prescription stimulants
   5. Methamphetamine
   6. Inhalants
   7. Sedatives or sleeping pills
   8. Hallucinogens
   9. Opioids
   10. Other
   11. None

C: Study CONSORT diagram.


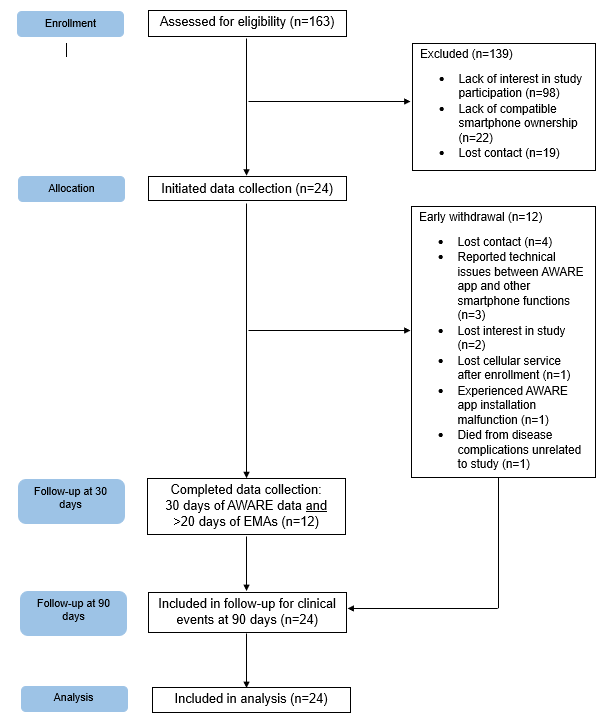


D: Comparison between baseline demographic and disease-related characteristics, as well as behavioral and psychological characteristics, for participants who completed the study (n=12) versus those who were withdrawn early (n=12). Unpaired t-tests have been used to compare score and range data, and Monte Carlo simulated chi-square tests were used to compare count data.

| **Characteristic** | **Total Complete (n=12)** | **Total Incomplete (n=12)** | **p** |
| --- | --- | --- | --- |
| Age in years | 50.0 (44.8, 57.3) | 44.5 (32.8, 57.3) | 0.297 |
| Male, n (%) | 10 (83.3) | 7 (58.3) | 0.389 |
| Race, n (%) |  |  | 0.576 |
| White | 11 (91.7) | 9 (75.0) |  |
| American Indian | 1 (8.3) | 2 (16.7) |  |
| Asian | ─ | 1 (8.3) |  |
| Education, n (%) |  |  | 1.000 |
| High school graduate or GED | 3 (25.0) | 3 (25.0) |  |
| Some college | 3 (25.0) | 3 (25.0) |  |
| College graduate | 5 (41.7) | 5 (41.7) |  |
| Post-college graduate degree | 1 (8.3) | 1 (8.3) |  |
| Employment, n (%) |  |  | 0.293 |
| Working for income | 7 (58.3) | 6 (50.0) |  |
| Retired | ─ | 3 (25.0) |  |
| Unemployed | 5 (41.7) | 3 (25.0) |  |
| Marital Status, n (%) |  |  | 0.595 |
| Never married | 2 (16.7) | 5 (41.7) |  |
| Married | 6 (50.0) | 4 (33.3) |  |
| Separated/Divorced | 4 (33.3) | 3 (25.0) |  |
| Presence of caregivers, n (%) | 7 (58.3) | 8 (66.7) | 1.000 |
| Body mass index | 31.1 (28.2, 33.4) | 28.7 (26.9, 31.6) | 0.345 |
| Baseline ALD stage, n (%) |  |  | 0.560 |
| Steatosis | 3 (25.0) | 3 (25.0) |  |
| Advanced fibrosis/cirrhosis | 2 (16.7) | ─ |  |
| Decompensated cirrhosis | 7 (58.3) | 8 (66.7) |  |
| Received transplantation | ─ | 1 (8.3) |  |
| History of alcohol-associated hepatitis, n (%) | 7 (58.3) | 6 (50.0) | 1.000 |
| Presence of ascites, n (%) | 5 (41.7) | 8 (66.7) | 0.430 |
| Presence of varices, n (%) | 3 (25.0) | 2 (16.7) | 1.000 |
| Presence of encephalopathy^a^, n (%) | 4 (33.3) | 2 (16.7) | 0.634 |
| Laboratory values |  |  |  |
| AST | 41.5 (35.3, 60.0) | 51.5 (39.8, 93.8) | 0.124 |
| ALT | 27.5 (24.8, 32.8) | 44.5 (29.0, 56.8) | 0.108 |
| Total bilirubin | 1.5 (1.0, 2.5) | 2.0 (0.7, 4.8) | 0.422 |
| Creatinine | 1.0 (0.8, 1.1) | 0.9 (0.8, 1.0) | 0.510 |
| INR | 1.1 (1.0, 1.4) | 1.3 (1.1, 1.4) | 0.931 |
| Sodium | 138.5 (135.3, 140.0) | 135.5 (131.8, 136.8) | 0.137 |
| MELD Score | 10.0 (8.5, 13.5) | 12.5 (8.0, 18.3) | 0.657 |
| Baseline AUDIT score^b^ | 21.0 (8.0, 27.5) | 26.5 (15.0, 28.3) | 0.261 |
| Interval of abstinence before study in days | 113.0 (10.75, 308.5) | 51.0 (14.0, 137.3) | 0.214 |
| Family history of AUD, n (%) | 9 (75.0) | 10 (83.3) | 1.000 |
| History of alcohol-related legal issues, n (%) | 7 (58.3) | 2 (16.7) | 0.094 |
| History of psychotherapy-based treatment for AUD^c^, n (%) | 7 (58.3) | 10 (83.3) | 0.386 |
| Active pharmacotherapy for AUD^d^, n (%) | 3 (25.0) | 3 (25.0) | 1.000 |
| History of comorbid psychiatric disorders, n (%) |  |  | 0.204 |
| Depression | 4 (33.3) | 7 (58.3) |  |
| Bipolar disorder | 2 (16.7) | ─ |  |
| Anxiety | 6 (50.0) | 5 (41.7) |  |
| Post-traumatic stress disorder | 2 (16.7) | ─ |  |
| Active pharmacotherapy for comorbid psychiatric disorders, n (%) | 7 (58.3) | 5 (41.7) | 0.682 |
| Nicotine use disorder, n (%) | 7 (58.3) | 8 (66.7) | 1.000 |
| Cannabis use disorder, n (%) | 4 (33.3) | 3 (25.0) | 1.000 |
| Opioid or other substance use disorder, n (%) | 1 (8.3) | 1 (8.3) | 1.000 |

*All continuous values are provided with median (IQR).

^a^Defined as West Haven Grade less than 3.

^b^One observation missing and not included in analysis.

^c^Defined as completion of either residential or outpatient treatment program.

^d^Defined as use of FDA-approved medications for AUD, including acamprosate, naltrexone, and disulfiram.

| **Questionnaire** | **Total Complete (n=12)** | **Total Incomplete (n=12)** | **p** |
| --- | --- | --- | --- |
| Patient Health Questionnaire (PHQ-9): Total Score, mean (SD) | 7.0 (5.2) | 6.4 (5.2) | 0.787 |
| Generalized Anxiety Disorder (GAD-7): Total Score, mean (SD) | 6.9 (5.5) | 5.2 (4.9) | 0.420 |
| Perceived Stress Scale: Total Score, mean (SD) | 16.7 (6.9) | 18.9 (5.4) | 0.871 |
| Connor-Davidson Resilience Scale (CD-RISC)-10: Total Score, mean (SD) | 28.4 (5.0) | 28.3 (5.4) | 0.969 |
| Perceived Social Support (F-SozU K-6): Mean Score, mean (SD) | 20.3 (4.9) | 25.6 (3.1) | 0.115 |
| General Self Efficacy Scale: Mean Score, mean (SD) | 29.9 (4.1) | 29.1 (5.5) | 0.322 |
| Insight Scale: Agree Response, n (%) |  |  | 1.000 |
| *I find many problems in my drinking.* | 10 (83.3) | 9 (75.0) |  |
| *I can control drinking any time if I want to.* | 4 (33.3) | 5 (41.7) |  |
| *All my problems can be solved only when I quit drinking.* | 5 (41.7) | 5 (41.7) |  |
| *My drinking did no harm to any member of the family.* | 2 (16.7) | 2 (16.7) |  |
| Readiness to change: Item Score, mean (SD) |  |  |  |
| *Importance* | 9.1 (2.4) | 9.2 (2.3) | 0.931 |
| *Confidence* | 8.9 (1.1) | 8.0 (2.2) | 0.209 |
| *Readiness to change* | 9.6 (0.9) | 9.2 (2.3) | 0.572 |
| Subjective Well-being: Item Score, mean (SD) | 6.8 (1.6) | 7.3 (2.0) | 0.493 |
| Chronic Liver Disease Questionnaire: Mean Score, mean (SD) |  |  |  |
| *Abdominal symptoms domain (1,5,17)* | 4.6 (1.6) | 5.2 (1.6) | 0.345 |
| *Fatigue domain (2,4,8,11,13)* | 3.4 (1.2) | 4.0 (1.4) | 0.283 |
| *Systemic domain (3,6,21,23,27)* | 4.3 (1.1) | 5.0 (1.3) | 0.170 |
| *Activity domain (7,9,14)* | 4.0 (0.9) | 4.7 (1.7) | 0.205 |
| *Emotional function domain (10,12,15,16,19,20,24,26)* | 4.3 (1.0) | 5.0 (1.0) | 0.093 |
| *Worry domain (18,22,25,28,29)* | 4.0 (1.5) | 4.1 (1.6) | 0.794 |
| *Overall score* | 4.1 (0.8) | 4.7 (1.1) | 0.144 |
| Brief Alcohol Craving Scale: Total Score, mean (SD) | 2.5 (1.9) | 2.8 (3.2) | 0.814 |

E: Pie charts illustrating relative quantity of each type of sensor data collected from each participant. Charts are additionally labeled with the type of phone (iPhone Operating System or Android); there are systematic differences in the amount of data collected depending on the type of phone being used.
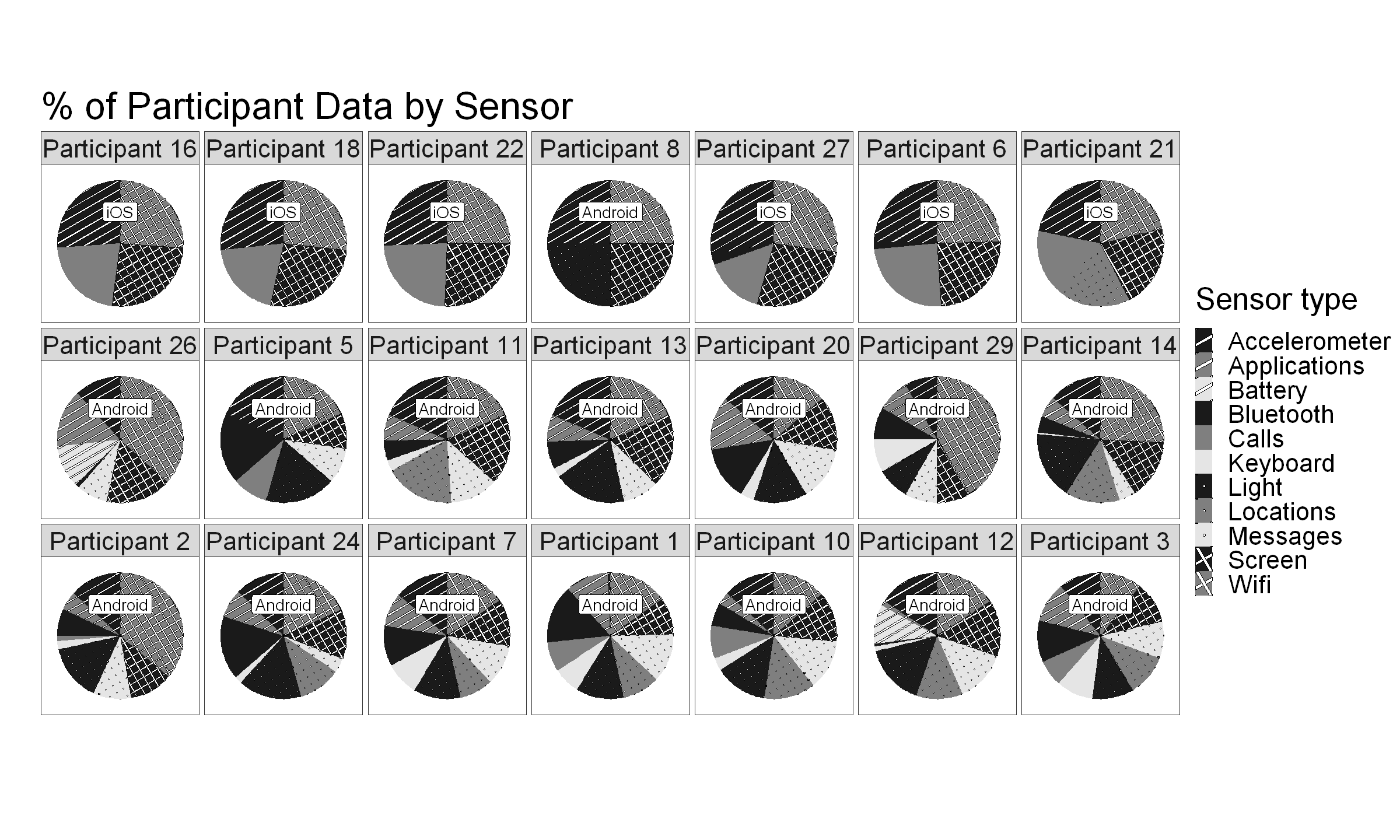


F: Plots of Daily Craving vs. Day for each of the participants with sufficient number of EMA responses. Missing data points reflect unanswered EMA surveys. Note that some participants exhibit a great deal of craving variance (e.g., Participant 6) while others do not (e.g., Participant 21).


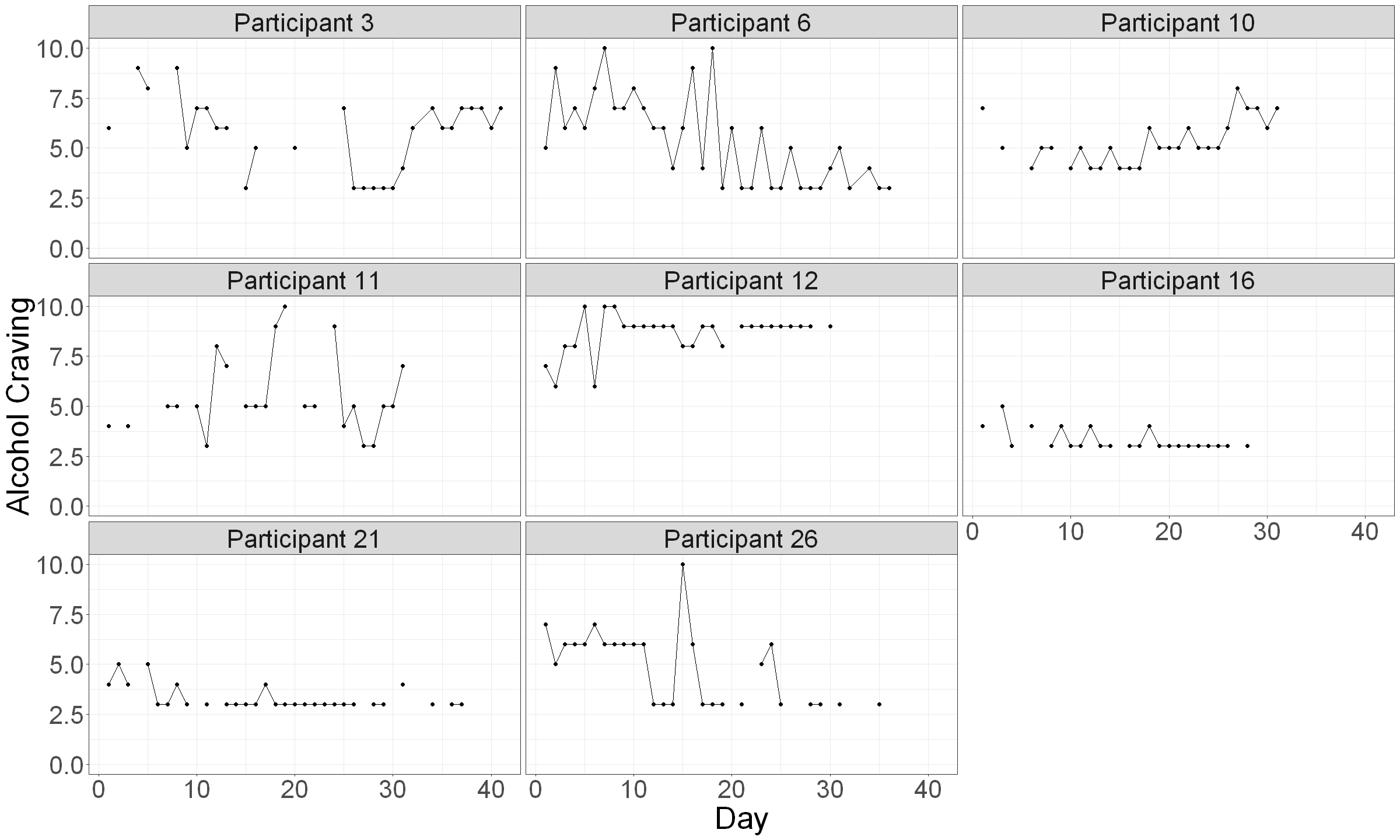


G. Sensors associated with craving, by subgroup comparing patients with alcohol-associated hepatitis (AH) and those without AH. Both features exhibit negative correlations, indicating that patients with AH exhibited lower accelerometer magnitudes.

| **Sensor Feature** | **Pearson *r* Difference** | **p** |
| --- | --- | --- |
| Accelerometer min magnitude | -0.203 | <0.001 |
| Accelerometer avg magnitude | -0.146 | 0.266 |

H. Change in CLDQ from baseline to follow-up among 12 participants who completed 30 days of study and follow-up visit. All p > 0.05 via independent two-sample *t*-tests.

| **Chronic Liver Disease Questionnaire: Mean Score, mean (SD)** | **Baseline** | **Follow-up** |
| --- | --- | --- |
| *Abdominal symptoms domain (1,5,17)* | 4.6 (1.6) | 4.7 (1.5) |
| *Fatigue domain (2,4,8,11,13)* | 3.4 (1.2) | 3.9 (0.8) |
| *Systemic domain (3,6,21,23,27)* | 4.3 (1.1) | 4.7 (0.8) |
| *Activity domain (7,9,14)* | 4.0 (0.9) | 4.8 (1.4) |
| *Emotional function domain (10,12,15,16,19,20,24,26)* | 4.3 (1.0) | 4.3 (1.0) |
| *Worry domain (18,22,25,28,29)* | 4.0 (1.5) | 4.6 (1.4) |
| *Overall score* | 4.1 (0.8) | 4.5 (0.8) |

I. Correlates of change in CLDQ and mood, by EMA responses.

| **Mood** | **Pearson *r* Correlation** | **T statistic** | **p** | **95% CI** |
| --- | --- | --- | --- | --- |
| Hopeful | 0.408 | 1.413 | 0.188 | (-0.217, 0.796) |
| Happy | 0.245 | 0.800 | 0.442 | (-0.382, 0.718) |
| Social support | 0.213 | 0.691 | 0.505 | (-0.411, 0.701) |
| Anxious | -0.189 | -0.609 | 0.556 | (-0.688, 0.432) |
| Angry | 0.185 | 0.596 | 0.565 | (-0.435, 0.686) |
| Afraid | 0.128 | 0.407 | 0.692 | (-0.481, 0.654) |
| Bored | 0.114 | 0.363 | 0.724 | (-0.492, 0.646) |
| Sad | -0.109 | -0.347 | 0.735 | (-0.643, 0.496) |
| Lonely | -0.084 | -0.267 | 0.795 | (-0.628, 0.515) |
| Calm | -0.049 | -0.155 | 0.880 | (-0.606, 0.540) |
| Stressed | 0.032 | 0.100 | 0.922 | (-0.552, 0.595) |

J. Correlates of change in CLDQ and sensors.

| **Sensor** | **Feature Description** | **Pearson r Correlation** | **T statistic** | **p** | **Parameter** | **95% CI** | **Adjusted p** |
| --- | --- | --- | --- | --- | --- | --- | --- |
| phone_keyboard_rapids_changeintextlengthequaltominusone | Decrease text length by 1 | 0.929 | 4.336 | 0.023 | 3 | (0.257, 0.995) | 0.937 |
| phone_keyboard_rapids_sessioncount | # typing sessions | 0.926 | 4.248 | 0.024 | 3 | (0.239, 0.995) | 0.937 |
| phone_locations_barnett_maxdiam | Largest distance between two locations | -0.803 | -2.692 | 0.055 | 4 | (-0.977, 0.025) | 0.937 |
| phone_screen_rapids_firstuseafter00unlock | Minutes until first unlock | -0.561 | -2.141 | 0.058 | 10 | (-0.858, 0.019) | 0.937 |
| phone_locations_barnett_probpause | Fraction of day stationary | 0.758 | 2.325 | 0.081 | 4 | (-0.139, 0.972) | 0.937 |
| phone_light_rapids_minlux | Minimum luminance | -0.917 | -3.247 | 0.083 | 2 | (-0.998, 0.372) | 0.937 |
| phone_light_rapids_medianlux | Median luminance | -0.904 | -2.990 | 0.096 | 2 | (-0.998, 0.435) | 0.937 |
| phone_locations_barnett_siglocsvisited | # significant locations visited | 0.721 | 2.079 | 0.106 | 4 | (-0.219, 0.967) | 0.937 |
| phone_locations_barnett_stdflightdur | Std dev of trips | -0.718 | -2.061 | 0.108 | 4 | (-0.966, 0.225) | 0.937 |
| phone_keyboard_rapids_changeintextlengthequaltoone | Increase in text length by 1 | 0.785 | 2.194 | 0.116 | 3 | (-0.317, 0.985) | 0.937 |
| phone_keyboard_rapids_averageinterkeydelay | Average interkey delay | -0.782 | -2.177 | 0.118 | 3 | (-0.985, 0.322) | 0.937 |
| phone_calls_rapids_outgoing_timefirstcall | Time of first outgoing call | 0.573 | 1.711 | 0.138 | 6 | (-0.221, 0.910) | 0.937 |
| phone_calls_rapids_incoming_timefirstcall | Time of first incoming call | 0.568 | 1.690 | 0.142 | 6 | (-0.228, 0.909) | 0.937 |
| phone_calls_rapids_outgoing_entropyduration | Outgoing call duration entropy | -0.567 | -1.685 | 0.143 | 6 | (-0.909, 0.230) | 0.937 |
| phone_calls_rapids_incoming_entropyduration | Incoming call duration entropy | -0.561 | -1.659 | 0.148 | 6 | (-0.907, 0.238) | 0.937 |
| phone_locations_barnett_maxhomedist | Max distance from home | -0.663 | -1.774 | 0.151 | 4 | (-0.959, 0.321) | 0.937 |
| phone_locations_barnett_rog | Radius of gyration | -0.661 | -1.763 | 0.153 | 4 | (-0.958, 0.324) | 0.937 |
| phone_calls_rapids_outgoing_minduration | Minimum outgoing call duration | 0.535 | 1.552 | 0.172 | 6 | (-0.272, 0.900) | 0.937 |
| phone_calls_rapids_incoming_sumduration | Total incoming call duration | -0.521 | -1.495 | 0.185 | 6 | (-0.897, 0.290) | 0.937 |
| phone_locations_barnett_disttravelled | Total distance traveled | -0.612 | -1.546 | 0.197 | 4 | (-0.951, 0.397) | 0.937 |
| phone_screen_rapids_mindurationunlock | Shortest screen unlock session | -0.398 | -1.371 | 0.200 | 10 | (-0.791, 0.228) | 0.937 |
| phone_accelerometer_rapids_minmagnitude | Minimum accelerometer magnitude | -0.398 | -1.371 | 0.200 | 10 | (-0.791, 0.228) | 0.937 |
| phone_calls_rapids_incoming_count | Incoming call count | -0.504 | -1.428 | 0.203 | 6 | (-0.892, 0.312) | 0.937 |
| phone_calls_rapids_incoming_distinctcontacts | # contacts incoming calls | -0.495 | -1.394 | 0.213 | 6 | (-0.889, 0.322) | 0.940 |
| phone_calls_rapids_outgoing_count | Outgoing call count | -0.485 | -1.359 | 0.223 | 6 | (-0.887, 0.333) | 0.944 |
| phone_calls_rapids_missed_timefirstcall | Time of first missed call | 0.477 | 1.331 | 0.232 | 6 | (-0.342, 0.885) | 0.944 |
| phone_calls_rapids_incoming_maxduration | Max incoming call duration | -0.461 | -1.271 | 0.251 | 6 | (-0.880, 0.361) | 0.960 |
| phone_screen_rapids_avgdurationunlock | Average screen unlock duration | -0.345 | -1.164 | 0.271 | 10 | (-0.767, 0.285) | 0.960 |
| phone_calls_rapids_outgoing_distinctcontacts | # contacts outgoing calls | -0.434 | -1.180 | 0.283 | 6 | (-0.872, 0.390) | 0.960 |
| phone_messages_rapids_sent_timefirstmessage | Time of first sent message | -0.517 | -1.207 | 0.294 | 4 | (-0.936, 0.508) | 0.960 |
| phone_applications_foreground_rapids_counteventemail | # of email app uses | 0.503 | 1.164 | 0.309 | 4 | (-0.521, 0.934) | 0.960 |
| phone_messages_rapids_received_timefirstmessage | Time of first received message | -0.496 | -1.143 | 0.317 | 4 | (-0.932, 0.528) | 0.960 |
| phone_accelerometer_rapids_medianmagnitude | Median accelerometer magnitude | -0.310 | -1.033 | 0.326 | 10 | (-0.751, 0.321) | 0.960 |
| phone_accelerometer_rapids_avgmagnitude | Average accelerometer magnitude | -0.310 | -1.030 | 0.327 | 10 | (-0.750, 0.321) | 0.960 |
| phone_screen_rapids_countepisodeunlock | # screen unlock events | 0.295 | 0.978 | 0.351 | 10 | (-0.335, 0.743) | 0.960 |
| phone_applications_foreground_rapids_timeoffirstuseall | Time of first use of any app | -0.462 | -1.041 | 0.357 | 4 | (-0.926, 0.559) | 0.960 |
| phone_locations_barnett_siglocentropy | Significant location entropy | 0.447 | 1.001 | 0.374 | 4 | (-0.572, 0.924) | 0.960 |
| phone_keyboard_rapids_changeintextlengthlessthanminusone | Decrease text length by less than 1 | 0.515 | 1.040 | 0.375 | 3 | (-0.673, 0.961) | 0.960 |
| phone_calls_rapids_incoming_meanduration | Mean incoming call duration | -0.363 | -0.955 | 0.376 | 6 | (-0.850, 0.459) | 0.960 |
| phone_messages_rapids_received_distinctcontacts | # contacts received messages | 0.440 | 0.981 | 0.382 | 4 | (-0.578, 0.922) | 0.960 |
| phone_accelerometer_rapids_stdmagnitude | Std dev accelerometer magnitude | -0.260 | -0.853 | 0.414 | 10 | (-0.726, 0.369) | 0.960 |
| phone_keyboard_rapids_changeintextlengthmorethanone | Increase text length by more than 1 | 0.471 | 0.926 | 0.423 | 3 | (-0.703, 0.956) | 0.960 |
| phone_bluetooth_rapids_countscansmostuniquedevice | # of scans of most frequently scanned Bluetooth device | -0.306 | -0.850 | 0.424 | 7 | (-0.806, 0.450) | 0.960 |
| phone_applications_foreground_rapids_timeoffirstusesocial | Time of first use of a social app | 0.573 | 0.988 | 0.427 | 2 | (-0.864, 0.989) | 0.960 |
| phone_calls_rapids_incoming_modeduration | Mode incoming call duration | -0.322 | -0.834 | 0.436 | 6 | (-0.837, 0.495) | 0.960 |
| phone_locations_barnett_hometime | Time spent at home | 0.391 | 0.850 | 0.443 | 4 | (-0.616, 0.913) | 0.960 |
| phone_applications_foreground_rapids_timeoflastuseemail | Time of last use of an email app | 0.442 | 0.855 | 0.456 | 3 | (-0.721, 0.953) | 0.960 |
| phone_calls_rapids_outgoing_sumduration | Total outgoing call duration | -0.290 | -0.742 | 0.486 | 6 | (-0.826, 0.521) | 0.960 |
| phone_locations_barnett_circdnrtn | Circadian routine | 0.354 | 0.758 | 0.491 | 4 | (-0.642, 0.905) | 0.960 |
| phone_applications_foreground_timeoffirstusetop1global | Time of first use of a frequently used app | -0.349 | -0.745 | 0.498 | 4 | (-0.904, 0.645) | 0.960 |
| phone_locations_barnett_wkenddayrtn | Circadian routine (weekend vs weekday) | 0.341 | 0.726 | 0.508 | 4 | (-0.650, 0.903) | 0.960 |
| phone_accelerometer_rapids_maxmagnitude | Max accelerometer magnitude | -0.200 | -0.645 | 0.533 | 10 | (-0.694, 0.422) | 0.960 |
| phone_keyboard_rapids_lastmessagelength | Last message length | 0.371 | 0.691 | 0.539 | 3 | (-0.760, 0.944) | 0.960 |
| phone_keyboard_rapids_maxtextlength | Max message length | 0.356 | 0.659 | 0.557 | 3 | (-0.767, 0.942) | 0.960 |
| phone_light_rapids_avglux | Average luminance | -0.438 | -0.689 | 0.562 | 2 | (-0.985, 0.903) | 0.960 |
| phone_calls_rapids_outgoing_countmostfrequentcontact | # outgoing calls to most frequent contact | -0.242 | -0.610 | 0.564 | 6 | (-0.809, 0.558) | 0.960 |
| phone_calls_rapids_outgoing_meanduration | Mean outgoing call duration | 0.230 | 0.580 | 0.583 | 6 | (-0.566, 0.804) | 0.960 |
| phone_wifi_connected_rapids_countscansmostuniquedevice | # scans of most used wifi network | -0.230 | -0.579 | 0.584 | 6 | (-0.804, 0.566) | 0.960 |
| phone_calls_rapids_outgoing_modeduration | Mode outgoing call duration | 0.224 | 0.563 | 0.594 | 6 | (-0.571, 0.802) | 0.960 |
| phone_wifi_connected_rapids_countscans | # wifi networks connected | 0.171 | 0.548 | 0.595 | 10 | (-0.447, 0.678) | 0.960 |
| phone_applications_foreground_rapids_timeoflastusesocial_media | Time of last use of a social media app | -0.286 | -0.517 | 0.641 | 3 | (-0.933, 0.798) | 0.960 |
| phone_applications_foreground_counteventtop1global | # uses of a frequently used app | 0.240 | 0.494 | 0.647 | 4 | (-0.710, 0.880) | 0.960 |
| phone_calls_rapids_outgoing_stdduration | Std dev outgoing call duration | -0.187 | -0.467 | 0.657 | 6 | (-0.788, 0.596) | 0.960 |
| phone_wifi_visible_rapids_uniquedevices | # wifi networks scanned | 0.219 | 0.448 | 0.677 | 4 | (-0.721, 0.875) | 0.960 |
| phone_messages_rapids_sent_timelastmessage | Time of last sent message | -0.219 | -0.448 | 0.677 | 4 | (-0.875, 0.721) | 0.960 |
| phone_applications_foreground_rapids_frequencyentropyall | Entropy of app use frequency | -0.210 | -0.430 | 0.689 | 4 | (-0.873, 0.725) | 0.960 |
| phone_applications_foreground_rapids_counteventall | # of uses of any app | 0.205 | 0.420 | 0.696 | 4 | (-0.727, 0.872) | 0.960 |
| phone_messages_rapids_received_timelastmessage | Time of last received message | -0.205 | -0.419 | 0.697 | 4 | (-0.872, 0.728) | 0.960 |
| phone_applications_foreground_rapids_countevententertainment | # of uses of entertainment apps | -0.201 | -0.409 | 0.703 | 4 | (-0.870, 0.730) | 0.960 |
| phone_wifi_connected_rapids_uniquedevices | # wifi networks connected | 0.121 | 0.385 | 0.708 | 10 | (-0.487, 0.650) | 0.960 |
| phone_keyboard_rapids_totalkeyboardtouches | Total keyboard touches | 0.229 | 0.407 | 0.711 | 3 | (-0.819, 0.924) | 0.960 |
| phone_applications_foreground_rapids_timeoflastuseall | Time of last use of any app | 0.188 | 0.382 | 0.722 | 4 | (-0.736, 0.867) | 0.960 |
| phone_locations_barnett_avgflightdur | Average trip duration | 0.187 | 0.380 | 0.723 | 4 | (-0.737, 0.867) | 0.960 |
| phone_keyboard_rapids_averagesessionlength | Average typing session length | 0.215 | 0.381 | 0.729 | 3 | (-0.824, 0.922) | 0.960 |
| phone_applications_foreground_rapids_timeoflastusetop1global | Time of last use of a frequently used app | 0.183 | 0.372 | 0.729 | 4 | (-0.738, 0.866) | 0.960 |
| phone_applications_foreground_rapids_timeoflastusesocial | Time of last use of a social app | -0.271 | -0.398 | 0.729 | 2 | (-0.977, 0.933) | 0.960 |
| phone_calls_rapids_outgoing_maxduration | Max outgoing call duration | -0.144 | -0.357 | 0.733 | 6 | (-0.771, 0.624) | 0.960 |
| phone_bluetooth_rapids_countscans | # Bluetooth devices scanned | -0.128 | -0.341 | 0.743 | 7 | (-0.730, 0.586) | 0.960 |
| phone_calls_rapids_missed_countmostfrequentcontact | # missed calls from most frequent contact | -0.135 | -0.333 | 0.751 | 6 | (-0.767, 0.630) | 0.960 |
| phone_applications_foreground_rapids_counteventsocial | # of uses of a social app | -0.161 | -0.327 | 0.760 | 4 | (-0.860, 0.748) | 0.960 |
| phone_calls_rapids_missed_timelastcall | Time of last missed call | 0.129 | 0.319 | 0.760 | 6 | (-0.633, 0.764) | 0.960 |
| phone_applications_foreground_rapids_counteventsocial_media | # of uses of a social media app | -0.160 | -0.324 | 0.762 | 4 | (-0.860, 0.749) | 0.960 |
| phone_light_rapids_maxlux | Maximum luminance | 0.234 | 0.341 | 0.766 | 2 | (-0.938, 0.976) | 0.960 |
| phone_light_rapids_stdlux | Std dev luminance | 0.229 | 0.332 | 0.771 | 2 | (-0.939, 0.975) | 0.960 |
| phone_calls_rapids_incoming_timelastcall | Time of last incoming call | -0.123 | -0.303 | 0.772 | 6 | (-0.762, 0.637) | 0.960 |
| phone_messages_rapids_sent_countmostfrequentcontact | # messages sent to most frequent contact | -0.144 | -0.292 | 0.785 | 4 | (-0.856, 0.756) | 0.960 |
| phone_messages_rapids_received_countmostfrequentcontact | # messages received from most frequent contact | -0.135 | -0.273 | 0.798 | 4 | (-0.853, 0.760) | 0.960 |
| phone_calls_rapids_missed_count | # missed calls | -0.108 | -0.266 | 0.799 | 6 | (-0.755, 0.646) | 0.960 |
| phone_applications_foreground_rapids_timeoffirstuseemail | Time of first use of an email app | 0.152 | 0.266 | 0.808 | 3 | (-0.843, 0.912) | 0.960 |
| phone_calls_rapids_missed_distinctcontacts | # contacts missed calls | -0.099 | -0.244 | 0.815 | 6 | (-0.751, 0.651) | 0.960 |
| phone_bluetooth_rapids_uniquedevices | # unique Bluetooth devices scanned | -0.080 | -0.211 | 0.839 | 7 | (-0.706, 0.617) | 0.972 |
| phone_applications_foreground_rapids_timeoffirstusesocial_media | Time of first use of a social media app | -0.107 | -0.187 | 0.864 | 3 | (-0.904, 0.856) | 0.972 |
| phone_messages_rapids_received_count | # received messages | 0.087 | 0.174 | 0.870 | 4 | (-0.780, 0.839) | 0.972 |
| phone_messages_rapids_sent_count | # sent messages | -0.084 | -0.168 | 0.875 | 4 | (-0.838, 0.781) | 0.972 |
| phone_calls_rapids_incoming_countmostfrequentcontact | # incoming calls from most frequent contact | -0.065 | -0.160 | 0.878 | 6 | (-0.736, 0.670) | 0.972 |
| phone_wifi_visible_rapids_countscans | # scanned wifi networks | -0.080 | -0.160 | 0.880 | 4 | (-0.837, 0.782) | 0.972 |
| phone_light_rapids_count | Light sensor count | 0.102 | 0.145 | 0.898 | 2 | (-0.952, 0.968) | 0.974 |
| phone_calls_rapids_incoming_minduration | Min incoming call duration | -0.051 | -0.126 | 0.904 | 6 | (-0.730, 0.678) | 0.974 |
| phone_screen_rapids_stddurationunlock | Std dev. screen unlock duration | -0.037 | -0.117 | 0.909 | 10 | (-0.598, 0.549) | 0.974 |
| phone_messages_rapids_sent_distinctcontacts | # contacts sent messages | -0.054 | -0.108 | 0.919 | 4 | (-0.829, 0.792) | 0.974 |
| phone_locations_barnett_avgflightlen | Average trip length | -0.035 | -0.069 | 0.948 | 4 | (-0.823, 0.799) | 0.985 |
| phone_calls_rapids_outgoing_timelastcall | Time of last outgoing call | -0.027 | -0.066 | 0.949 | 6 | (-0.718, 0.691) | 0.985 |
| phone_screen_rapids_sumdurationunlock | Total screen unlock duration | -0.017 | -0.055 | 0.957 | 10 | (-0.585, 0.562) | 0.985 |
| phone_locations_barnett_stdflightlen | Std dev of flight length | -0.015 | -0.030 | 0.977 | 4 | (-0.817, 0.806) | 0.989 |
| phone_calls_rapids_incoming_stdduration | Std dev incoming calls | -0.011 | -0.026 | 0.980 | 6 | (-0.710, 0.699) | 0.989 |
| phone_screen_rapids_maxdurationunlock | Longest screen unlock duration | 0.002 | 0.008 | 0.994 | 10 | (-0.572, 0.576) | 0.994 |
| phone_applications_foreground_counteventcom.facebook.moments | # of Facebook uses | NA | NA | NA | 4 | NA | NA |
| phone_applications_foreground_counteventcom.twitter.android | # of Twitter uses | NA | NA | NA | 4 | NA | NA |
| phone_applications_foreground_rapids_counteventdating | # of uses of a dating app | NA | NA | NA | 4 | NA | NA |
